# Community and bystander interventions for the prevention of suicide: Protocol for a systematic review

**DOI:** 10.1371/journal.pone.0270375

**Published:** 2022-06-30

**Authors:** Asha Worsteling, Byron W. Keating

**Affiliations:** 1 QUT Business School, Queensland University of Technology, Brisbane, QLD, Australia; 2 UQ School of Psychology, The University of Queensland, Brisbane, QLD, Australia; Xiamen University - Malaysia Campus: Xiamen University - Malaysia, MALAYSIA

## Abstract

Suicide has a wide reaching and devastating impact on society. This article presents a study protocol for a systematic review of the literature on community and bystander interventions to reduce the incidence of suicide. These interventions are focused on people other than the person at-risk and are designed to increase awareness of warning signs and knowledge of how to most effectively respond. While there have been many studies undertaken on community and bystander intervention programs, we lack a synthesis of evidence regarding how an effective program is created and implemented. The proposed systematic review will address this gap by presenting the first comprehensive review on this topic. The specific aims of the review are to: (1) determine whether community and bystander interventions effectively increase bystander action to prevent suicide and, if so; (2) to understand what creates an effective community suicide intervention. The insights gathered will inform policy and guide investment in better evidence-based suicide interventions for the future.

## Introduction

Suicide is one of the biggest killers globally. Over 700,000 people die of suicide every year, and it is the fourth leading cause of death for people between the ages of 15 and 29 [1]. 77% of suicides occur in low to middle-income countries, making it a global issue to be tackled [1]. Between 10 to 30 more suicide attempts per suicide are predicted, although this figure is rarely accurately reported because most suicide attempts go undocumented [2]. This enormous number of suicide attempts gravely impacts the mental health and quality of life of the person attempting suicide as well as those close to them. Furthermore, a previous suicide attempt is one of the most significant predictors of a future deaths by suicide [1]. Despite attempts to reduce the number of suicides, the rate of decrease is stagnating, giving rise to the need for further research into effective prevention methods.

### Description of the interventions

Research thus far has suggested that suicide interventions should use a combination of nine strategies simultaneously to generate the best result. The nine strategies include: providing follow-up care for those who have attempted suicide, providing evidence-based treatment for at-risk individuals, equipping primary care providers to better recognise and support at-risk individuals, improving the competence of first-responders to deal with a suicidal crisis, mental health education in schools and organizations, training the community to identify warning signs and respond accordingly, community awareness programs, responsible media reporting, and reducing access to lethal means of suicide [3].

Notwithstanding the collective importance of these strategies, some have greater potential than others. For example, prior research indicates that only around 20–30% of individuals had any contact with health professionals before attempting suicide [4,5], whereas 70–90% communicated warning signs to friends or family [6,7]. This emphasizes the need to better understand those strategies targeting people other than those at risk. Warning signs can be behavioural (e.g., withdrawing or preparing a will), verbal (e.g. saying “I can’t do this anymore”), or situational (e.g. recent break-up) [8].

Community intervention programs aim to increase awareness of these warning signs, increase knowledge of how to intervene and improve attitudes towards suicide [9,10]. The World Health Organisation [1] acknowledges that one of the most significant obstacles to reducing the rates of suicide is the stigma associated with suicide and mental illness as a whole as it deters people who need help from seeking it. It is possible that such stigma may also deter people from acting when confronted by an at-risk person. As such, improving attitudes towards suicide and increasing awareness of mental illness should be one of the first steps to engaging the community in preventing suicide.

Another barrier to preventing suicide is that, as mentioned above, most at-risk individuals will communicate warning signs to peers but not medical professionals. However, the public is underprepared for this role as most people are not able to identify warning signs, and few know what to do when given knowledge of a potential suicide [8,11]. As such, it is vital to educate the public about suicide so they can intervene effectively.

Lastly, community suicide education programs aim to increase confidence and intention to intervene [12]. Community suicide education programs can be delivered via a variety of mediums; most commonly through one to four-hour lecture or workshop formats but can also be delivered online through websites or interactive apps and in print media such as posters, newspapers, and handbooks [10]. Interventions are delivered in various contexts, often within schools or universities, as well as workplaces, and to people in specific geographic locations [10].

### How the intervention might work

Community suicide interventions aim to encourage bystander action in the event of a crisis. The difficulty with bystander action is that many people are usually present in an emergency, which makes it difficult to determine who is most responsible to act [13]. A recent study found that one of the biggest reasons people do not intervene in a suicide attempt is that they thought someone else closer to the individual would take action [14]. Inaction resulting from diffusion of responsibility is known as the bystander effect [13]. The bystander intervention model (BIM) is a potential mechanism for overcoming the bystander effect. It suggests five processes are necessary for bystander intervention; noticing the critical situation, interpreting the situation as an emergency or urgent, assuming personal responsibility to help, feeling confident and competent in helping, and reaching a conscious decision to help [11]. By educating the public on warning signs of suicide, they are more likely to interpret the situation and interpret it as critical. By educating them on what to do to intervene, the public feels more confident intervening. By increasing their intention to intervene, they are more likely to take responsibility for intervening and make the conscious decision to help. As such, community suicide education programs can overcome the bystander effect and increase the likelihood of intervention in a crisis or emergency.

### Why is it important to do this review?

Suicide globally causes an enormous number of preventable deaths. Bystanders are uniquely positioned to help prevent these deaths if they know what to do. While there have been many studies completed on community and bystander intervention programs, to date we lack a synthesis of evidence regarding how an effective program is created and implemented [10,12]. In particular, we need to know how best to engage and educate bystanders. The findings of this systematic review are expected to lead to better-informed suicide prevention programs, many of which are funded and/or delivered by public and not-for-profit organizations with limited resources.

### Objectives

This review has two objectives: (1) to determine whether community and bystander interventions effectively increase bystander action to prevent suicide and, if so, (2) to understand what creates an effective community suicide intervention.

## Materials and methods

The protocol for this systematic review has been registered with PROSPERO (CRD42022304647). The protocol was developed following the recommendations of the Cochrane Handbook for Systematic Reviews of Interventions [15] and the guidelines of the Preferred Reporting Items for Systematic Review and Meta-Analysis (PRISMA-P) [16]. The recommendations from these two key sources are synthesized into a four-step process.

### Step 1: Criteria for selecting studies

The criteria used for the selection of studies will be based on the PICO (Population, Interventions, Comparators, Outcomes) framework. Each component of this framework, including explicit reference to inclusion and exclusion criteria, is discussed below.

#### Types of participants (population)

Bystanders of all ages and sexes will be included in the review. As we are focused on the impact of interventions on the behavior of the general public; first responders, doctors, psychologists, and other medical professionals will be excluded from the review.

#### Types of interventions (interventions)

Interventions to be included in the review will be those described as either community, bystander, or peer interventions, or those that include general public education in at least one of the following domains:

- Increases knowledge of warning signs of suicide
- Increases knowledge of actions to intervene (e.g., alerting emergency services)
- Increases intention to intervene
- Increases confidence to intervene
- Improves attitudes towards suicide and reduces stigma.

The intervention should be able to be delivered at scale to a community or large group such as a school, workplace, town or city, or any other similar group or region. Interventions targeting groups of medical professionals or first responders will be excluded.

#### Types of comparisons (comparators)

All types of experimental and quasi-experimental study designs will be considered in the review, including randomized and non-randomized trials and studies using before-and-after comparison. Studies will be included regardless of publication status or the language of publication, however, studies without a well-detailed description of the control or before-and-after comparison will be excluded. A well-detailed description is defined as a description that would allow for replication. Studies with a control can use either an inactive control (e.g., no treatment) or an active control (e.g., different variations of the same intervention).

#### Types of outcome measures (outcomes)

Primary outcome measures include the following:

- Knowledge of warning signs; assessed using any valid measure, including a survey testing knowledge or an experimental scenario testing ability to recognise a person at-risk.
- Knowledge of how to intervene; assessed using any valid measure. For example, a survey of appropriate responses to different at-risk behaviours or participant behavioural responses to experimental scenarios.
- Attitudes towards suicide; measured using any valid scale of attitudes.
- Secondary outcome measures include the following:
- The theory used to implement the intervention, e.g. psychological distance theory or the bystander intervention model.
- Intention to intervene; measured using any valid scale or experimental scenario testing behaviour.
- Confidence to intervene; measured using any valid scale.

#### Ethical considerations

The search will be limited to studies that were undertaken in line with accepted ethical standards. To support this condition, we will contact authors of studies that do not explicitly report information indicating that the research was peer-reviewed by an appropriately constituted ethics committee or institutional review board.

### Step 2: Search methods for identification of studies

#### Sources to search

We will conduct a tailored search of each of the following bibliographic databases from inception to the date of search:

- Web of Science
- PsycINFO
- CENTRAL
- EMBASE
- Google Scholar

These databases were identified as they include a broad range of journals that publish content related to the objectives of this review. There will be no restrictions on the date, language or publication status applied to the searches. The search strategy will also include a search for grey literature that meets this same standard via the following sources:

- Open grey (opengrey.eu)
- Grey Literature Report (New York Academy of Medicine; www.greylit.org).

We will also review the reference lists of included studies and any relevant systematic reviews for any other potentially eligible studies. We will contact the authors of papers if any clarifications are required or for relevant unpublished data mentioned within the article.

#### Designing the search strategy

The search process will begin with a search of the relevant databases for terms related to the objectives of the study. For example, the search algorithm will focus initially on the keywords “suicide” AND “intervention,” and scope-based limiters associated with “bystander” AND “peer” OR “community.” Filters associated with the PICO framework will also be used to narrow and refine the search. For example, where available, the search results could be filtered based on system provided keywords that would help to target an appropriate population, type of experimental approach, or exposure variable.

### Step 3: Data collection

Data will be collected according to the recommendations of the Cochrane Handbook for Systematic Reviews of Interventions [15] and the PRISMA-P guidelines [16]. This guidance extends to the screening of studies, procedures for data extraction and management, and assessment of risk and bias. These will be undertaken with the assistance of the Covidence systematic review software.

#### Screening the studies

Review authors will independently screen the titles and abstracts of all records identified through the search strategy. Abstracts at this stage will be marked as potentially relevant or not. The full text of all potential studies will then be independently assessed for inclusion by the review authors, and exclusion of any study will be justified. Any disagreements will be resolved through discussion. We will present the results of our study selection process in a PRISMA flow diagram.

#### Data extraction and management

Review authors will design a data extraction form that will be piloted on a small number of studies and modified if necessary. Two review authors will independently extract data from included studies. Where data is missing, the authors of the study will be contacted. We will extract the following characteristics:

- Study aims
- Context: location of the intervention, time period of the intervention, funding sources.
- Participants: total number, mean age, gender distribution, occupation, previous exposure to suicide.
- Method of the intervention: e.g., lecture, poster, or app, duration, mode of recruitment of people to the intervention.
- Goals of the intervention: e.g., attitude improvement or knowledge building.
- Theory: any theory used to design the intervention (e.g., BIM)
- Study design: type and method of data collection (e.g., survey).
- Outcomes: where available, outcome data of all primary and secondary outcomes and any other outcomes measured in the study and the time points at which data was collected.
- Ethics: was information provided on the ethics review process and/or outcome.

#### Assessment of risk of bias in included studies

Review authors will independently assess risk of bias in all included studies, and any disagreements will be resolved by discussion. We will assess risk of bias according to the recommendations of the Cochrane Handbook for Systematic Reviews of Intervention [15]. The following domains will be assessed for bias in randomised trials:

1. Bias arising from the randomisation process: was the sequence generation random or imbalanced? Was allocation concealed?
2. Bias due to deviations from intended interventions: were participants blind to their allocation, and were there any deviations to intended intervention that arose due to the context, adherence, or implementation?
3. Bias due to missing outcome data: was any missing outcome data adequately addressed?
4. Bias in measurement of the outcome: was measurement appropriate, consistent and were outcome assessors blind to participants condition?
5. Bias in selection of reported results: is there any selective reporting of data, or is there any suggestion in the way results are reported?

The following domains will be assessed for bias in non-randomised trials and quasi-experiments:

1. Bias due to confounding: are there any confounding variables that predict the intervention and the outcome of interest?
2. Bias in selection of participants into the study: does the exclusion of some participants lead to a possible association between the intervention and the outcome of interest?
3. Bias in classification of interventions: could the intervention status have been misclassified?
4. Bias due to deviations from intended interventions: are there systematic deviations in the intervention to what was intended?
5. Bias due to missing data: is there significant missing data and can this data be attributed to a prognostic factor?
6. Bias in measurement of the outcome: were there any errors in measuring outcome data?
7. Bias in selection of reported result: was there any selective reporting of results?

Each domain will be assessed as low or high risk of bias or having some concerns. If any domain in a study is assessed as high risk of bias, the overall risk of bias for the study will be high. If a study has several domains with some concerns, then the overall risk of bias will also be high. We will present the risk of bias assessments in the summary table as part of the GRADE assessment.

### Step 4: Data analysis

#### Outcome measurement

Dichotomous data will be analysed using risk ratios (RRs) with 95% confidence intervals (95% CIs). As there are likely to be a range of continuous outcome measures across studies, we will calculate a standardised mean difference (SMD) and their 95% confidence intervals (95% CIs).

#### Unit of analysis issues

If any issues with the unit of analysis in a study are identified, the study’s author will be contacted for further information and the results re-analysed. We will follow the methodology suggested by the EPOC analytic methods group and the Cochrane Handbook for Systematic Reviews of Interventions [15] to avoid any unit of analysis issues. If identified, we will include cluster randomised trials, adjust their sample sizes or standard errors, and analyse their results along with individually randomised trials.

#### Dealing with missing data

We will contact authors for any missing data or statistics (e.g. standard deviations). If we are unable to obtain the missing information, the missing statistics will be calculated using the methods outlined in the Cochrane Handbook for Systematic Reviews of Interventions [15]. Missing data due to attrition will be considered part of the risk of bias assessment and will be noted in the summary table and considered in the discussion of results.

#### Assessment of heterogeneity

We will inspect confidence intervals on forest plots for the degree of overlap to determine the potential direction and magnitude of heterogeneity. We will quantify inconsistencies using the I^2^ and Chi^2^ test statistics. As it is predicted that there may be a large amount of heterogeneity between studies due to methodological diversity and diversity in the interventions, many factors will be involved in interpreting heterogeneity. The Cochrane Handbook for Systematic Reviews of Interventions [15] suggests using the following benchmarks for the interpretation of I^2^:

1. unimportant: 0% to 40%
2. moderate: 30% to 60%
3. substantial: 50% to 90%
4. considerable: 75% to 100%.

We will assess I^2^ with relevance to these benchmarks. However, we will also take into consideration the results of the Chi^2^ test and the magnitude and direction of effects.

#### Assessment of reporting biases

If ten or more studies are included in the systematic review, a funnel plot will be created and visually assessed for any evidence of asymmetry. We will use the Egger test to explore any asymmetry further, recognising that there can be other reasons for funnel plot asymmetry than reporting bias.

### Data synthesis

We aim to conduct a meta-analysis if at least two studies examine any outcome that is sufficiently similar (i.e., has no substantial heterogeneity as determined by I^2^ and Chi^2^). As it is expected that there will be considerable heterogeneity, a random-effects model will be used for the meta-analysis. If a meta-analysis is not feasible due to the variety of interventions being included, we will do a narrative synthesis of the results.

#### Subgroup analysis and investigation of heterogeneity

One of the present review’s hypotheses is that community suicide education programs differ in effectiveness across participants of different levels of responsibility (e.g., gatekeepers vs peers). As such, to determine how different levels of responsibility/closeness to the at-risk individual plays a role, we will conduct subgroup analysis in the following domains:

- People in a position of authority (e.g., teachers or managers in workplaces)
- Types of connection between people (e.g., people who may have close or weak ties to others)
- Different organizational settings (e.g., people within schools or workplaces)
- Different localities (e.g., interventions designed for a whole city/town).

We also aim to determine which specific elements of the community interventions lead to the best outcomes for improvements in intention to intervene, attitudes, and knowledge of suicide. As such, we will conduct subgroup analysis in the following types of interventions:

- Workshop or lecture-based interventions
- Technology-based interventions (e.g., apps or websites)
- Hard copy-based interventions (e.g., posters or information booklets).

If multiple studies have used the same theory to inform their intervention, we will also conduct a subgroup analysis of these interventions to determine if certain theories lead to a more favourable outcome. Additionally, we will consider performing a subgroup analysis on any additional dimensions where there is sufficient data, and where we consider that they may be relevant to the outcome of the interventions.

#### Sensitivity analysis

We plan to carry out the following sensitivity analysis for the primary outcomes to determine the effect on results of methodological choices or assumptions:

- We will remove studies with a high risk of bias and compare the result against the main result to determine the effect of study quality on the results.
- We will remove studies with imputed data and compare the result against the main result to determine the effect of our methodological assumptions on the overall results.
- We will remove studies with dropout rates at higher than 20% to determine the effect of attrition bias on the review results.

For completeness, we will also consider combinations of the above criteria and remove studies where the results do not compare favorably with the main results.

#### Summary of findings and assessment of the certainty of the evidence

We will provide results from all included studies in a summary table. We will also include a rating of confidence in the quality of evidence for all studies within this table. The ratings of methodological rigor will be based on the GRADE approach outlined in the Cochrane Handbook for Systematic Reviews of Interventions [15]. Review authors will independently assess the quality of evidence and disagreements will be resolved by discussion. All decisions to downgrade the quality of evidence will be justified using footnotes, and judgments will be incorporated into the discussion of results.

## Discussion

This article presents a study protocol for a systematic review of the literature on community and bystander interventions to reduce the incidence of suicide. To our knowledge this is the first comprehensive review on the topic. The specific aims of the review are to: (1) determine whether community and bystander interventions effectively increase bystander action to prevent suicide and, (2) to understand what creates an effective community suicide intervention. Using a four-step process, the resulting systematic review will gather the data to resolve these questions and inform policy and investment in better evidence-based suicide interventions for bystanders in the future.

As with all research, this protocol and the proposed systematic review have some inherent limitations that need to be considered when interpreting and relying on the findings. For example, the protocol indicates that the scope of the review will be limited to certain databases, to certain bystander categories, and only considers experimental and quasi-experimental studies. While these decisions are strategic and will help to strengthen the quality of the evidence and the trustworthiness of the findings, it does mean that we are purposefully excluding observation studies, and qualitative and conceptual research. Using this search strategy also means we cannot guarantee that interesting and impactful insights will not be missed. We do not believe that these limitations, however, will lessen the relevance of the proposed protocol, particularly considering the comprehensive literature search, and the timeliness and importance of the research topic.

## Supporting information

S1 Table PRISMA-P 2015 checklist. (DOCX)
